# Beyond Relapses: A Multimodal Biomarker Framework for Progression Independent of Relapse Activity and Smouldering Multiple Sclerosis

**DOI:** 10.3390/brainsci16070735

**Published:** 2026-07-12

**Authors:** Nayeli Alejandra Sánchez-Rosales, Edgar Ricardo Valdivia-Tangarife, Blanca Miriam Torres-Mendoza, Antonio Kobayashi-Gutiérrez, Francisco Javier Frías-Márquez, Betsabe Contreras-Haro, Martha Rocío Hernández-Preciado, Ana Miriam Saldaña-Cruz, Enrique Gomez-Figueroa, José De Jesús García-Rivera, Teresita J. Villaseñor-Cabrera, Miriam E. Jiménez-Maldonado, Fabiola González-Ponce, Jazmin Marquez-Pedroza

**Affiliations:** 1 Departamento de Neurología, Unidad de Alta Especialidad Médica (UMAE), Centro Médico Nacional de Occidente (CMNO), Instituto Mexicano Del Seguro Social (IMSS), Guadalajara 44340, Jalisco, Mexico; naye_ale@hotmail.com (N.A.S.-R.); franciscojfrias@hotmail.com (F.J.F.-M.); jesusgarcianeuro@gmail.com (J.D.J.G.-R.); 2 Departamento de Neurociencias, Centro Universitario de Ciencias de La Salud (CUCS), Universidad de Guadalajara, Guadalajara 44340, Jalisco, Mexico; teresita.villasenor@academicos.udg.mx (T.J.V.-C.); elizabeth.jimenez@academicos.udg.mx (M.E.J.-M.); 3 Departamento de Disciplinas Filosóficas, Metodológicas e Instrumentales, Centro Universitario de Ciencias de la Salud (CUCS), Universidad de Guadalajara, Guadalajara 44340, Jalisco, Mexico; bltorres1@hotmail.com (B.M.T.-M.); mrociohp@hotmail.com (M.R.H.-P.); 4 División de Neurociencias, Centro de Investigación Biomédica de Occidente (CIBO), Instituto Mexicano Del Seguro Social (IMSS), Guadalajara 44340, Jalisco, Mexico; drkoba@hotmail.com; 5 Departamento de Medicina Interna, Unidad de Alta Especialidad Médica (UMAE), Centro Médico Nacional de Occidente (CMNO), Instituto Mexicano Del Seguro Social (IMSS), Guadalajara 44340, Jalisco, Mexico; 6 Departamento de Ciencias Biomédicas, Centro Universitario Tonalá, Universidad de Guadalajara, Tonalá 45425, Jalisco, Mexico; betsabecoha@gmail.com; 7 Unidad de Investigación Biomédica 02, Instituto Mexicano Del Seguro Social (IMSS), Guadalajara 44340, Jalisco, Mexico; 8 Unidad Médica de Alta Especialidad, Hospital Pediátrico, Centro Médico Nacional de Occidente (CMNO), Instituto Mexicano Del Seguro Social (IMSS), Guadalajara 44340, Jalisco, Mexico; 9 Instituto de Terapéutica Experimental y Clínica, Departamento de Fisiología, Centro Universitario de Ciencias de La Salud (CUCS), Universidad de Guadalajara, Guadalajara 44340, Jalisco, Mexico; ana.saldanac@academicos.udg.mx (A.M.S.-C.); fgponce.ln@gmail.com (F.G.-P.); 10 Hospital Civil de Guadalajara Fray Antonio Alcalde, Guadalajara 44280, Jalisco, Mexico; enrique.g.figueroa@gmail.com

**Keywords:** multiple sclerosis, progression independent of relapse activity, smouldering MS, multimodal biomarker score, paramagnetic rim lesions, serum GFAP, optical coherence tomography, digital biomarkers

## Abstract

**Highlights:**

**What are the main findings?**

- Four complementary biomarker domains—advanced MRI (paramagnetic rim lesions), serum GFAP/NfL, retinal OCT (GCIPL thinning), and digital step-count metrics—each capture a distinct facet of the smouldering biology that drives progression independent of relapse activity (PIRA).
- Combining serum GFAP and NfL identifies patients at markedly higher PIRA risk (HR 4.71, 95% CI 2.05–9.77), showing the added value of integrating markers over any single measure.

**What are the implication of the main findings?**

- The proposed Multimodal PIRA Score (MPS) is a conceptual framework for future risk stratification, monitoring, and clinical-trial enrichment in smouldering MS.
- It requires prospective, competing-risks validation before any clinical use, defining a clear roadmap for future research.

**Abstract:**

Progression independent of relapse activity (PIRA) and smouldering multiple sclerosis (MS) represent major unmet challenges in contemporary MS care. Disability may accumulate independently of clinical relapses, driven in part by chronic compartmentalised inflammation behind a relatively intact blood–brain barrier and incompletely captured by conventional monitoring tools. This narrative review synthesises evidence across four complementary biomarker domains for detecting smouldering MS and PIRA: advanced MRI (paramagnetic rim lesions [PRLs], slowly expanding lesions, deep grey matter atrophy, quantitative susceptibility mapping); fluid biomarkers (serum glial fibrillary acidic protein [sGFAP], serum neurofilament light chain [sNfL]); retinal optical coherence tomography (ganglion cell–inner plexiform layer thinning); and digital health metrics (wearable accelerometry, digital Symbol Digit Modalities Test). In a single prospective cohort, combined elevation of sGFAP and sNfL conferred a 4.71-fold increased hazard for PIRA (HR 4.71; 95% CI 2.05–9.77); independent data suggest sGFAP may carry selectivity for progression beyond sNfL, although this remains to be confirmed. No single domain sufficiently characterises smouldering pathology. We therefore propose a hypothesis-generating Multimodal PIRA Score (MPS) as a conceptual validation scaffold intended to structure—rather than inform—prospective multicentre evaluation against a long-horizon disability endpoint. Harmonisation of acquisition protocols, reference ranges, and digital phenotyping algorithms remains a prerequisite.

## 1. Introduction

Multiple sclerosis (MS) affects more than 2 million individuals worldwide and is the leading non-traumatic cause of neurological disability in young adults [1]. Highly effective disease-modifying therapies (DMTs) substantially suppress relapses and gadolinium-enhancing lesions, yet disability continues to accrue in a large proportion of patients despite apparent radiological stability—the clinical–radiological paradox [2]. In a pooled analysis of 1656 participants from the OPERA I and OPERA II trials, the majority of confirmed disability worsening occurred independently of clinical relapses, a phenomenon termed progression independent of relapse activity (PIRA) [3]. Subsequent real-world analyses confirmed that PIRA accounts for at least half of all disability accrual events in relapsing–remitting MS, occurs early in the disease course, and portends substantially worse long-term outcomes [4,5,6,7].

These observations have prompted a reconceptualisation of MS as a dual-process disease. The term smouldering *MS* refers to a treatment-refractory component characterised by chronic microglial and innate immune activation within the central nervous system. This process persists behind a relatively intact blood–brain barrier, is only partially captured by conventional MRI, and contributes to diffuse cortical demyelination, trans-synaptic neurodegeneration, deep grey matter atrophy, and progressive axonal energy failure, independently of new focal inflammatory lesions [8,9,10]. This results in gradual disability accumulation that may occur independently of relapses, contributing to the clinico-radiological paradox in MS [2,5].

Identifying reliable biomarkers of smouldering disease is therefore an important clinical priority. Four complementary domains have emerged as candidate components of a conceptual PIRA framework: advanced MRI markers of chronic lesional pathology and grey matter loss; fluid biomarkers of astrocytic activation and neuroaxonal injury; retinal optical coherence tomography (OCT) as a non-invasive marker of neurodegeneration; and digital health measures of real-world functional change. No single domain is likely to fully capture this complex process, and integrating these measures may provide a useful direction for future research [8,9].

Scope and contribution. Single-domain evidence for these markers has been consolidated in recent consensus statements and reviews [8,9,10,11]. This review does not present new data. Its contribution is twofold. First, it makes explicit the cross-domain integration logic—mapping the partially orthogonal biological facets that each domain captures, and identifying where the domains are redundant rather than complementary. Second, it sets out a transparent and falsifiable validation roadmap (including competing-risks methodology, endpoint selection, and external validation in under-represented Latin American populations) for moving from isolated single-domain associations to a prospectively validated composite. The Multimodal PIRA Score introduced below is offered strictly as a conceptual scaffold to structure that effort, not as a validated instrument for individual treatment decisions.

In this review, PIRA refers to confirmed disability worsening independent of clinical relapses, although definitions vary across studies [6]. Given this conceptual heterogeneity and the absence of standardised outcomes, a narrative synthesis was considered the most appropriate methodological approach. This narrative review was designed as a hypothesis-generating conceptual synthesis, in line with recent narrative reviews and consensus statements in this field [9,10], and therefore does not follow PRISMA guidelines.

### Search Strategy

PubMed/MEDLINE and EMBASE were searched (last updated April 2026) using controlled vocabulary and free-text terms: “multiple sclerosis” AND (“progression independent of relapse activity” OR “PIRA” OR “smouldering MS” OR “paramagnetic rim lesions” OR “serum GFAP” OR “serum neurofilament” OR “optical coherence tomography” OR “digital biomarkers” OR “disability worsening”). Inclusion criteria were peer-reviewed original research articles, reviews, and consensus statements published in English between January 2000 and April 2026; studies of human MS populations; and articles addressing at least one of the four biomarker domains (advanced MRI, fluid biomarkers, retinal OCT, or digital health metrics) in relation to PIRA, smouldering MS, disability progression, or confirmed disability worsening. Within these, prospective cohort studies, randomised controlled trials, meta-analyses, and observational studies with sample size greater than 50 were prioritised. Exclusion criteria were case reports, conference abstracts, editorials and non-peer-reviewed commentary, preprints, non-English publications, and purely preclinical studies without translational relevance. Titles and abstracts were screened for relevance, and full texts of potentially eligible articles were reviewed. Final inclusion was determined by author consensus based on methodological quality, relevance to PIRA biomarker research, and the currency of the evidence. Reference lists of key articles were also hand-searched to identify additional relevant studies. A total of 52 references were retained. As this was a hypothesis-generating narrative synthesis, no formal risk-of-bias assessment, quantitative pooling, or PRISMA flow diagram was undertaken. The evidence base is not exhaustive and may be subject to selection and publication bias, as discussed in Section 5.3.

## 2. Pathophysiological Basis of PIRA and Smouldering Disease

### 2.1. The Two-Hit Model of MS Disability

**Figure 1:**
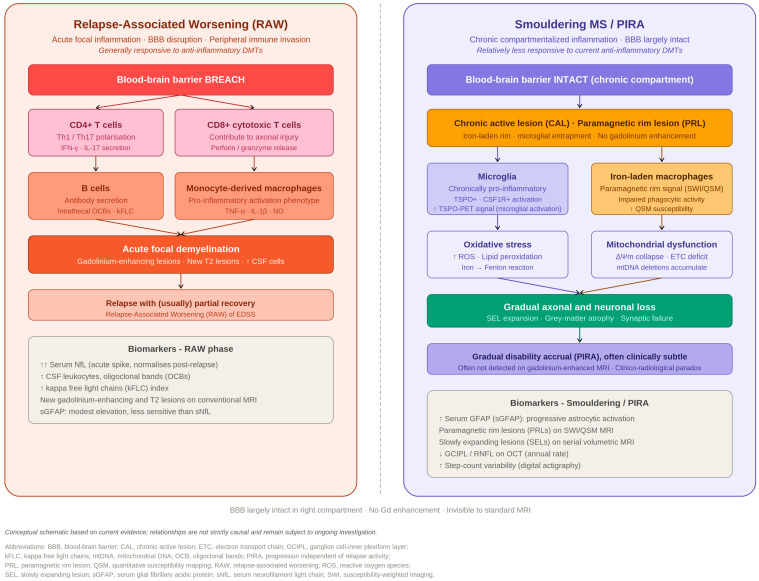
Pathophysiology of relapse-associated worsening (RAW) versus smouldering MS and PIRA. (**Left panel**) RAW is driven by peripheral lymphocyte invasion across a breached blood–brain barrier, with acute focal demyelination, CD4+ (Th1/Th17) and CD8+ T-cell activation, B-cell recruitment with intrathecal OCB and kFLC synthesis, and monocyte-derived macrophage activation; characteristic biomarkers include acute sNfL spikes, CSF leukocytosis, new gadolinium-enhancing and T2 lesions, and modest sGFAP elevation. (**Right panel**) Smouldering MS/PIRA is driven by chronic compartmentalised innate immune activation behind a largely intact blood–brain barrier, with chronic active (paramagnetic rim) lesions harbouring iron-laden macrophages and activated microglia, oxidative stress and mitochondrial failure, slow axonal and neuronal loss, SEL expansion, grey-matter atrophy, and clinically silent disability accrual. The schematic is conceptual, and the depicted relationships are not strictly causal. Abbreviations: BBB, blood–brain barrier; ETC, electron transport chain; OCB, oligoclonal bands; ROS, reactive oxygen species; SWI, susceptibility-weighted imaging; other abbreviations as in Table 1.

Contemporary neuropathological and neuroimaging evidence supports a two-hit model of disability accumulation. The first hit—focal demyelination from peripheral lymphocyte invasion across a disrupted blood–brain barrier—produces relapse-associated worsening (RAW) and responds to approved anti-inflammatory DMTs [12,13]. The second hit—diffuse compartmentalised neurodegeneration driven by innate immune activation within the CNS—produces PIRA and is largely refractory to current anti-inflammatory therapeutics [14,15]. Both mechanisms coexist and potentiate each other through excitotoxic glutamate release, persistent sodium-channel dysregulation, and mitochondrial energy failure in demyelinated axons [15,16] (Figure 1).

### 2.2. Microglial Activation and Chronic Active Lesions

Smouldering pathology is histopathologically dominated by chronic activation of microglia and macrophages at the rim of white matter lesions, perpetuating a cycle of demyelination and axonal injury [14,15]. Iron released from myelin degradation generates toxic reactive oxygen species that propagate oxidative damage to adjacent axons [17]; mitochondrial DNA deletions accumulate in demyelinated axons, further compromising energy supply [18]; and the resulting energy failure triggers reversal of the sodium–calcium exchanger, initiating a cascade of axonal degeneration that occurs silently over years [16]. This histopathological substrate corresponds in vivo to paramagnetic rim lesions (PRLs) on susceptibility-sensitive MRI and to slowly expanding lesions (SELs) on longitudinal volumetric analysis [19,20].

It is important to note, however, that the in vivo correlates of smouldering inflammation remain under active debate and should not be treated as settled. The assumption that the paramagnetic phase rim itself indexes ongoing inflammatory activity has been challenged: recent imaging work indicates that the presence of a paramagnetic phase rim may be influenced by lesion age and temporal evolution and should not be interpreted simplistically as a direct and exclusive marker of chronic active inflammation [21]. Moreover, PRLs and SELs should not be regarded as interchangeable readouts of a single ‘chronic active’ substrate; in a direct comparison they represented largely *non-overlapping* lesion populations [22], implying that they capture partially distinct biological processes. These caveats are directly relevant to the use of PRL count as a marker of smouldering disease and are revisited in Section 4.2, where component selection for the proposed score is discussed (Section 5.2).

### 2.3. Diffuse Neurodegeneration

Beyond focal white matter lesions, MS features diffuse grey matter pathology including cortical demyelination, synaptic loss, and trans-synaptic neurodegeneration mediated by axonal degeneration from white matter lesion burden [12,13]. Deep grey matter structures—particularly the thalamus—suffer disproportionate atrophy as relay nuclei for multiple cortical and subcortical pathways [23]. Diffuse microglial activation in apparently normal-appearing white matter further drives tissue injury beyond what is visible on conventional T2-weighted sequences, contributing to the clinico-radiological paradox [8,10,14].

## 3. Multimodal Biomarker Domains

### 3.1. Advanced MRI

PRLs are susceptibility-weighted MRI markers of iron-rimmed chronic active lesions without gadolinium enhancement. In observational cohorts, a higher PRL burden has been independently associated with an increased risk of five-year EDSS worsening and may add prognostic information on disability accrual beyond total T2 lesion volume [19,24,25]. It should be emphasised that this association has been demonstrated principally against broad disability-worsening outcomes rather than against a strictly relapse-independent (PIRA-specific) endpoint, and that PRL assessment is currently limited by inter-reader variability, dependence on field strength (typically 3T or higher), and the abPRL count should therefore be regarded as a candidate marker of chronic lesional pathology, with its specificity for smouldering disease requiring further validation. PRL burden may generate false-positive signals when rims primarily reflect lesion age or chronicity rather than ongoing inflammatory activity. Conversely, limited MRI availability, variable acquisition protocols, and imperfect lesion detection may lead to underestimation of PRL burden.

SELs, identified by longitudinal volumetric analysis as lesions showing perilesional volume expansion, represent ongoing perilesional tissue destruction and are under investigation as candidate imaging endpoints for trials of smouldering-targeting agents [20,22]. Because SELs and PRLs largely do not co-localise [22], they are treated here as distinct—rather than equivalent—imaging readouts. Deep grey matter, particularly thalamic, atrophy constitutes one of the strongest MRI correlates of long-term disability [23]. Quantitative susceptibility mapping (QSM) provides non-invasive quantification of paramagnetic iron at lesion rims and can identify the subset of chronic lesions with an inflammatory rim, correlating with rim-associated tissue damage [26]; however, QSM remains limited in clinical availability, lacks an agreed normal threshold, and is not yet validated against PIRA. For these reasons, QSM is discussed as a promising research tool but is not incorporated into the proposed score. Table 1 summarises all biomarker domains, including the specific outcome against which each has been evaluated.

### 3.2. Fluid Biomarkers

Serum neurofilament light chain (sNfL) is associated with confirmed disability worsening and reflects DMT pharmacodynamic effects [27,28]. However, its ability to distinguish PIRA from RAW is limited because it rises with acute focal inflammation and is influenced by age, body mass index, and systemic comorbidities. Meaningful individual-level interpretation therefore requires appropriate age-adjusted reference ranges [27,28,29,30].

Serum glial fibrillary acidic protein (sGFAP) reflects astrocytic structural disruption and reactive gliosis, processes thought to contribute to the smouldering pathological state. In the prospective Swiss MS Cohort (Meier et al. 2023; 355 patients, of whom 252 formed the B-cell depleting cohort in which the time-to-event analysis was performed), the *combined* elevation of sGFAP and sNfL conferred a 4.09-fold increased hazard for confirmed disability worsening (HR 4.09; 95% CI 2.04–8.18; *p* < 0.001) and a 4.71-fold increased hazard for PIRA (HR 4.71; 95% CI 2.05–9.77; *p* < 0.001) [31]. These hazard ratios reflect the joint effect of both markers and therefore do not, by themselves, establish a property of sGFAP in isolation. Two findings suggest that sGFAP provides information beyond sNfL alone. First, in the same cohort, the combination of low sGFAP with high sNfL did not confer increased PIRA risk (HR 1.17; 95% CI 0.34–4.10), indicating that the elevated risk was not driven by sNfL alone [31]. Second, in an independent prospective relapsing–remitting MS cohort, baseline sGFAP was the only fluid or imaging biomarker significantly elevated in patients who subsequently developed PIRA, and an sGFAP concentration above 65 pg/mL was associated with higher odds of PIRA (odds ratio 4.3; 95% CI 1.44–12.86; *p* = 0.009) [32]. The additive value of combined sGFAP and sNfL monitoring for PIRA prediction—wherein a single elevated marker alone did not significantly raise PIRA risk—has since been reproduced in an extended B-cell depleting cohort from the same consortium [33].

Taken together, these findings support sGFAP as a promising candidate fluid biomarker that may provide information relevant to PIRA, rather than as a definitively validated PIRA-specific marker. The available evidence is based on a limited number of cohorts, predominantly involving White European populations, and the clinical applicability of absolute sGFAP thresholds has not been prospectively established across populations or assay platforms. This selectivity is not uniform across study settings: in a post hoc analysis of the ASCEND trial in secondary-progressive MS without acute inflammatory activity (n = 264), neither baseline sGFAP nor its longitudinal change was prognostic of, or dynamically associated with, confirmed disability progression over two years, suggesting that the prognostic value of sGFAP may depend on disease stage, follow-up duration, and residual inflammatory activity [34]. Independent studies have also reported broader associations between elevated sGFAP and progressive MS phenotypes CSF kappa free light chains [35,36,37]. 

### 3.3. Retinal Optical Coherence Tomography

GCIPL thinning on spectral-domain OCT reflects global retinal ganglion-cell neurodegeneration mediated by retrograde trans-synaptic degeneration [38]. Lower retinal thickness is independently associated with disability progression risk, even after adjustment for relapse history and MRI lesion volume [38,39,40,41]; as with PRLs, this evidence pertains principally to disability-worsening outcomes rather than to a strictly PIRA-anchored endpoint. OCT is non-invasive, widely available, and highly reproducible, and longitudinal monitoring is clinically feasible [41]. A recognised confounder is the floor effect after optic neuritis, and prior optic neuritis can bias eye-level measurements. Prior optic neuritis and advanced retinal damage may reduce the sensitivity of GCIPL thinning to detect further neurodegeneration because of floor effects, potentially producing false-negative results. Retinal abnormalities unrelated to MS may also complicate interpretation [38,41].

The inner nuclear layer (INL) thickens transiently during relapses, likely reflecting microcystic macular oedema associated with neuroinflammation [42]. This behaviour is approximately orthogonal to GCIPL atrophy and makes the INL a candidate marker for distinguishing neuroinflammatory (RAW-related) from neurodegenerative retinal change [42]. Because the INL indexes acute inflammatory activity rather than the smouldering process, it is not incorporated into the progression-oriented score proposed below, but it is retained conceptually as a potential RAW-discriminating signal that future work may integrate.

### 3.4. Digital Health Metrics

Wearable accelerometry captures real-world ambulatory function with ecological validity and temporal granularity that episodic clinic-based scales cannot match [43,44]. In a single prospective cohort, continuous daily step-count monitoring showed a strong inverse association with EDSS (*p* < 0.001), with patients with progressive MS averaging on the order of approximately 2500 fewer steps per day than those with relapsing MS (*p* < 0.01) [43]; the precise magnitude should be interpreted cautiously given the single-cohort source and inter-device variability. Importantly, this evidence relates step count to EDSS and MS phenotype rather than to a validated PIRA endpoint, and step-count variability is included below as a real-world functional readout whose specific predictive value for PIRA remains to be established [43,44]. Step-count variability may also produce false-positive signals because it can be influenced by device characteristics, occupational and lifestyle patterns, fatigue, mood, musculoskeletal conditions, environmental factors, and temporary illness.

Weekly, digitally administered SDMT via smartphone enables more frequent ecological cognitive sampling than episodic clinic visits and reduces the floor and ceiling effects of fixed-interval assessment; high adherence to digital SDMT has been demonstrated [45,46]. It must be stated plainly that the available digital SDMT evidence concerns feasibility, adherence, and satisfaction—not predictive validity for PIRA—and that no study has yet validated the digital SDMT against a relapse-independent disability endpoint [45,46]. Passive phone phenotyping (deriving cognitive and mobility surrogates from GPS patterns, keyboard dynamics, and device usage) offers additional low-burden endpoints, but standardisation of digital outcome algorithms across platforms remains a prerequisite for regulatory acceptance [44]. The digital SDMT is therefore presented as a feasibility-supported candidate measure rather than as an evidence-based PIRA predictor.

## 4. Proposed Multimodal PIRA Score (Conceptual)

### 4.1. Rationale for Integration

**Figure 2:**
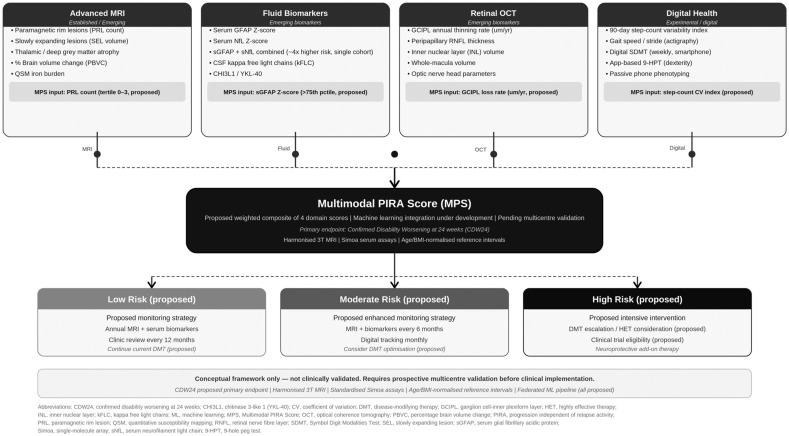
Conceptual Multimodal PIRA Score (MPS) framework. Four complementary biomarker domains feed into a composite score: (i) advanced MRI (PRL count, scored by tertiles) [19,22,24,25]; (ii) fluid biomarkers (sGFAP Z-score above the 75th age-adjusted percentile) [31,32,35,36]; (iii) retinal OCT (annualised GCIPL thinning rate, scored by quartiles) [39,40,41]; and (iv) digital health (90 day step-count coefficient of variation, scored by quartiles) [43,44,46]. Domain scores are summed to a maximum of 10 and categorised into illustrative tiers: low (0–3), moderate (4–6), and high (7–10). This conceptual, hypothesis-generating framework has not been prospectively validated; its components, weights, thresholds, and tier-based actions are illustrative placeholders and should not be used for individual clinical decision-making. A single maximal domain should prompt clinical review irrespective of the total score (Section 4.3). Abbreviations as in Table 1.

No single biomarker domain adequately characterises smouldering MS in isolation. The four domains capture partially orthogonal biological facets: imaging localises the anatomical substrate of chronic lesional pathology; fluid biomarkers reflect molecular astrocytic and axonal consequences; OCT provides a cumulative neuroaxonal readout; and digital metrics capture real-world functional impact. A composite score therefore offers a theoretically attractive route to risk stratification [11,47] (Figure 2). We stress that this rationale motivates the hypothesis, not the validity, of any specific composite; the construction below is illustrative and is intended to be tested, refined, or rejected by prospective data.

### 4.2. Component Selection and the Rationale for One Marker per Domain

Within each domain, we propose one feasible marker for the conceptual score, recognising that prospective validation may modify these choices. Selection was guided by two principles: (i) relative specificity for smouldering rather than acute relapse-related processes, and (ii) practical applicability across heterogeneous, including resource-constrained, centres. PRL count was selected over SELs and QSM because it can be assessed cross-sectionally from a single susceptibility-weighted acquisition, whereas SEL detection requires serial, computationally intensive volumetric MRI and QSM has limited availability and no agreed threshold. This choice prioritises feasibility, although PRL count retains the interpretive limitations discussed above [21,22,24]. sGFAP was selected over sNfL because sNfL is less specific for PIRA than RAW, and over kFLC because kFLC is invasive, primarily diagnostic, and unsuitable for serial monitoring [27,31,32,37]. GCIPL thinning was selected as a cumulative neurodegenerative measure, whereas INL was excluded because it primarily reflects acute inflammation, although it remains a candidate marker for distinguishing RAW [41,42]. Step-count variability was selected as the digital measure most directly associated with disability and supported by feasibility data [43,44]. Prospective studies should assess whether adding excluded markers—SELs, QSM, sNfL, INL, or digital SDMT—improves discrimination.

### 4.3. Score Structure and Explicit Treatment of Its Limitations

As an illustrative example, the MPS is structured as a simplified additive system integrating four domain scores: (i) PRL count, scored by tertile (0–3); (ii) sGFAP, scored as a Z-score above the 75th age-adjusted percentile (0–1); (iii) GCIPL annualised thinning rate, scored by quartile (0–3); and (iv) 90-day step-count coefficient of variation, scored by quartile (0–3). Domain scores are summed to a maximum of 10. This follows established practice for simplified clinical prediction rules, in which continuous predictors are categorised into ordered tiers and coefficients are coarsened into integer weights for interpretability [48]. Several design choices are provisional. First, categorising continuous predictors inevitably sacrifices predictive information relative to models that preserve continuous distributions [48]. Second, the integer ranges shown are not equal across domains (PRL 0–3, sGFAP 0–1, GCIPL 0–3, step-count 0–3), which imposes an implicit—and unintended—unequal weighting; this is internally inconsistent with the notion of equal domain contribution, and we make explicit that true domain weights must be derived empirically rather than asserted. Third, the maximum of 10 and the tier cut-points are arbitrary in the conceptual phase. These limitations are acknowledged to make the framework concrete and testable. Prospective validation should determine whether fully parameterised Cox or competing-risks models, which preserve continuous predictor effects and derive empirical weights from time-to-event data, provide better discrimination and calibration.

A single-domain override may be necessary because an additive score can mask a marked abnormality in one domain. Under the illustrative scheme, a patient with a maximal score in one domain and no abnormalities in the others would still be classified as low risk. Therefore, a maximal value in any domain should prompt individual clinical review regardless of the composite score. This limitation further supports the need for empirically derived weighting and competing-risks modelling.

### 4.4. Risk Tiers and Clinically Coherent Actions

Illustrative risk tiers are pragmatically defined as low (MPS 0–3), moderate (MPS 4–6), and high (MPS 7–10). These thresholds are provisional and serve only to illustrate the principle of risk stratification. Their final definition should be based on prospective, data-driven calibration against a pre-specified relapse-independent outcome.

Any actions linked to these tiers should remain consistent with the biology that the score is intended to capture. Because smouldering disease may be insufficiently addressed by currently approved anti-inflammatory DMTs, a high score should not automatically lead to escalation of these therapies. If prospectively validated, a low score may support routine or less frequent monitoring; a moderate score may prompt closer review and reassessment of overall disease control; and a high score may warrant intensified monitoring, shared decision-making, and consideration of enrolment in clinical trials evaluating therapies directed at smouldering biology, such as BTK inhibitors or microglial modulators. Thus, the principal near-term value of a validated MPS would be as a prognostic-enrichment and monitoring tool, particularly for identifying candidates for clinical trials, rather than as a routine treatment-decision instrument.

### 4.5. Endpoint Selection for Validation

**Figure 3:**
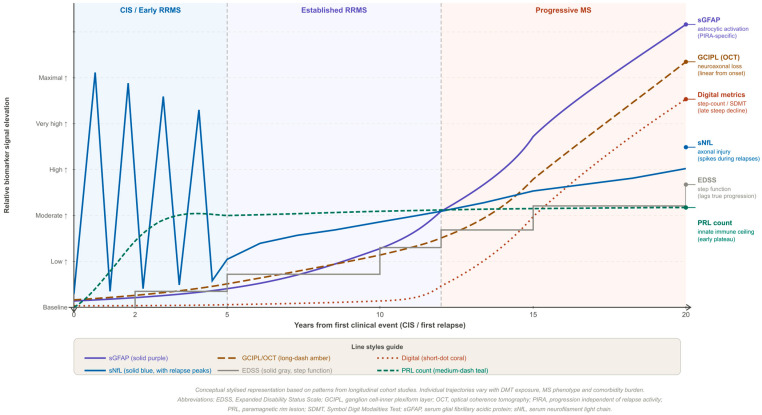
Conceptual temporal evolution of multimodal biomarkers across the MS disease course. This stylised, hypothesis-generating illustration depicts the relative trajectories of six biomarkers from clinically isolated syndrome through relapsing–remitting MS to the progressive phase over approximately 20 years. sGFAP is shown as progressively increasing; GCIPL thinning as approximately linear neuroaxonal loss; digital metrics as declining later in the disease course; sNfL as showing relapse-associated spikes with later chronic elevation; EDSS as lagging behind biological progression; and PRL count as rising early and then plateauing. These trajectories are illustrative only, are not empirical data or validated temporal models, and should not be used for individual clinical interpretation or decision-making. Individual trajectories may vary according to DMT exposure, phenotype, comorbidities, and other clinical factors. Abbreviations as in Table 1.

The choice of confirmed disability worsening at 24 weeks (CDW24) as a validation endpoint is pragmatic but imperfectly matched to the biology the score targets. Smouldering-driven progression accrues over years (Figure 3 depicts an approximately 20-year horizon), whereas a 24-week confirmation window preferentially captures relatively rapid, step-wise worsening—that is, change closer to RAW than to the slow accumulation the framework seeks to isolate. A short-horizon endpoint therefore risks both under-detecting slow smouldering accrual and conflating it with RAW. We propose that validation should privilege longer-horizon confirmation (for example, sustained worsening confirmed at ≥48 weeks or over multiple years) and/or slope-based or continuous disability metrics (for example, the slope of EDSS over time or composite measures such as EDSS-Plus), with explicit, pre-specified separation of PIRA events from RAW. CDW24 may be retained as a secondary, pragmatic anchor for comparability with existing trials, but should not be the sole primary endpoint for a score designed to capture slow progression.

### 4.6. Validation Framework

Validation of the MPS should address both discrimination and calibration. Discrimination, commonly assessed using the concordance index (c-statistic or AUC), indicates how well the model ranks patients according to predicted risk. Calibration evaluates the agreement between predicted and observed outcome rates [48]. Because disability worsening is a time-to-event outcome that may occur in the context of relapse-associated worsening, validation should use Fine–Gray competing-risks regression or cause-specific Cox models rather than logistic regression. Model performance may also be summarised using measures such as the index of predictive accuracy, which combines discrimination and calibration on a 0–10 scale [48]. The MPS should be compared with a null model and with existing predictors, such as sGFAP alone, PRL burden, or EDSS trajectory, to determine whether combining biomarker domains provides incremental prognostic value. 

### 4.7. Machine Learning Integration

The four-domain structure is amenable to machine-learning augmentation. Federated-learning architectures enable privacy-preserving multicentre model development without centralising patient data. Integration of HLA-DRB1*15:01 genotype, serum CHI3L1/YKL-40, and emerging neuroimmune markers may further personalise risk prediction [49,50]. Domain weights in this preliminary proposal are intentionally left to be determined: prospective data will be required to derive empirically validated weights, and penalised survival models or federated neural networks may perform substantially differently from the illustrative additive scheme [51].

**Table 1:** Summary of multimodal biomarker domains for PIRA and smouldering MS: mechanisms, the specific outcome against which each marker has been evaluated, and key limitations. Reference numbers correspond to citations in the main text.

Domain	Biomarker	Mechanism	Reported Association (Outcome Assessed)	Key Limitation
Advanced MRI	Paramagnetic rim lesions (PRLs)	Chronic microglial activation; iron-laden macrophages at lesion rim	Higher burden independently associated with increased risk of 5-year EDSS worsening (broad disability outcome, not PIRA-specific) [19,24,25]; rim partly reflects lesion age [21]	Inter-reader variability; ≥3T dependent; no standardised threshold; contested biological meaning
Advanced MRI	Slowly expanding lesions (SELs)	Ongoing perilesional demyelination beyond visible lesion edge	Candidate imaging endpoint under investigation for smouldering-MS trials; largely non-overlapping with PRLs [20,22]	Serial volumetric MRI
Advanced MRI	Deep grey matter atrophy	Trans-synaptic degeneration from WM lesion burden	Among strongest MRI correlates of long-term disability; thalamic atrophy most predictive (general disability) [23]	Confounded by treatment-related brain volume changes, including pseudo-atrophy [52]
Advanced MRI	QSM iron burden	Iron in rim macrophages; identifies inflammatory rim subset	Identifies inflamed chronic lesions; correlates with rim damage [26]; not validated against PIRA	Limited availability; no agreed normal threshold; not in MPS
Fluid	Serum NfL (sNfL)	Axonal cytoskeletal release; acute spikes, chronic plateau	Associated with confirmed disability worsening; reflects DMT efficacy [27,28]	Less PIRA-specific than sGFAP; elevated by ageing/comorbidities [29,30]
Fluid	Serum GFAP (sGFAP)	Astrocytic structural disruption; reactive gliosis	Combined sGFAP + sNfL: ~4–5 × PIRA hazard (HR 4.71; B-cell depleting cohort, n = 252 of a 355-patient study); low-sGFAP/high-sNfL not elevated (HR 1.17) [31]. sGFAP alone (>65 pg/mL) associated with PIRA in an independent cohort (OR 4.3) [32]	Selectivity relative, few cohorts; age-dependent ranges lacking; insensitive to acute relapses [31,32,35]
Fluid	CSF kFLC index	Intrathecal B-cell immunoglobulin synthesis	Diagnostic marker (high sensitivity for CIS/MS) [37]; This has not yet been demonstrated to predict PIRA.	Invasive; unsuitable for serial monitoring; diagnostic not prognostic
Retinal OCT	GCIPL annual thinning rate	Retinal ganglion-cell neurodegeneration; trans-synaptic loss	Baseline/longitudinal thinning independently associated with long-term disability worsening (not PIRA-specific) [39,40,41]	Floor effect and confound after optic neuritis [38,41]
Retinal OCT	Inner nuclear layer (INL)	Transient oedema during acute inflammation	Orthogonal to GCIPL; candidate marker to distinguish RAW from PIRA [42]	Indexes acute inflammation; excluded from MPS by design
Digital health	Step-count variability (90-day)	Real-world ambulatory function; gait impairment	Strong inverse association with EDSS (single cohort, *p* < 0.001); ~2500 fewer steps/day in progressive vs. relapsing MS (*p* < 0.01); not validated against PIRA [43]	Device heterogeneity; no standardised algorithm; single-cohort [43,44]
Digital health	Digital SDMT (weekly smartphone)	Real-world cognitive processing speed	Feasibility/adherence demonstrated only; predictive validity for PIRA not established [45,46]	Learning effect; depression confound; validation gap vs. pen-and-paper [45,46]

**Abbreviations:** CIS, clinically isolated syndrome; CSF, cerebrospinal fluid; DMT, disease-modifying therapy; EDSS, Expanded Disability Status Scale; GCIPL, ganglion cell–inner plexiform layer; INL, inner nuclear layer; kFLC, kappa free light chains; MPS, Multimodal PIRA Score; OCT, optical coherence tomography; PIRA, progression independent of relapse activity; PRL, paramagnetic rim lesion; QSM, quantitative susceptibility mapping; RAW, relapse-associated worsening; SDMT, Symbol Digit Modalities Test; SEL, slowly expanding lesion; sGFAP, serum glial fibrillary acidic protein; sNfL, serum neurofilament light chain; WM, white matter.

## 5. Discussion

### 5.1. Interpretation of Synthesised Evidence

This review identifies PRLs, sGFAP, GCIPL thinning, and step-count variability as candidate components of a multimodal PIRA biomarker panel, while recognising the uneven strength of the available evidence and the heterogeneity of the outcomes assessed. The possibility that sGFAP may provide information relevant to progression is supported by the lack of an observed excess PIRA risk among participants with high sNfL but low sGFAP, as well as an independent association between baseline sGFAP and subsequent PIRA. However, these findings remain limited by the small number of available cohorts and should not be interpreted as establishing sGFAP as a PIRA-specific biomarker. The observed association is biologically plausible given the role of reactive astrogliosis in chronic active lesion pathology [14,31,32]. This potential selectivity is relative, supported by only a small number of cohorts, and should be interpreted cautiously. The potential complementarity of OCT-derived GCIPL thinning and fluid biomarkers is conceptually relevant: GCIPL thinning provides a structural measure of cumulative neuroaxonal loss and may be less directly influenced by acute inflammatory activity, whereas sGFAP and sNfL may fluctuate with disease activity. Wearable step-count variability adds a real-world, system-level dimension inaccessible to imaging or fluid markers. The four domains are therefore plausibly complementary rather than redundant, which is the conceptual basis for integration—though this complementarity remains a hypothesis to be tested rather than an established property [38,43] (Figure 3).

A central interpretive caveat concerns the heterogeneity of the outcomes against which the component markers have been validated. The synthesised evidence relates different markers to different endpoints—PIRA in some studies, but confirmed disability worsening, five-year EDSS worsening, or general disability progression in others (Table 1). Aggregating markers validated against different criteria into a single composite that is then presented as a PIRA-specific predictor is not straightforward, and the discrepancy between the constructs underlying the input evidence and the construct the score purports to predict must be treated as a first-order limitation rather than a detail. This consideration reinforces the need for prospective validation against a single, pre-specified, relapse-independent endpoint. Consistent with this, studies that have measured several markers together report that they are often only weakly correlated and may index partly different disease processes, underscoring both their potential complementarity and the difficulty of combining them naively.

### 5.2. Clinical Implications

If prospectively validated, the proposed framework could have implications for future MS practice beyond trial design. In patients with apparent radiological stability and low relapse rates—frequently considered clinically stable under current paradigms—a high composite score could flag covert smouldering disease and provide a quantitative basis for closer monitoring and, where appropriate, evaluation for a smouldering-targeted clinical trial before disability becomes irreversible. Conversely, a consistently low score could support extended monitoring intervals, reducing unnecessary clinical burden in genuinely stable patients. This is consistent with an emerging treat-to-target philosophy in which therapeutic adequacy is judged not only by suppression of relapses and new MRI lesions but by arrest of the smouldering component [11]. Implementation would require training in multi-domain biomarker interpretation, standardised reporting, and decision-support integrated within electronic health records. As emphasised above, a high score should not be read as a mandate to escalate anti-inflammatory therapy that is not expected to modify smouldering biology. The LACTRIMS region is well positioned to contribute cross-population cohort data, given the under-representation of non-European populations in current biomarker evidence, which consensus statements have highlighted as a key limitation [9,11].

### 5.3. Limitations

Several limitations qualify this synthesis. As a narrative review, it presents no new data and is subject to selection and publication bias; cohort studies reporting null associations between biomarker domains and PIRA may be under-represented in the peer-reviewed literature. Definitional heterogeneity for PIRA across studies—varying observation windows, anchor events, and EDSS thresholds—limits cross-cohort comparability, and the markers reviewed have been validated against heterogeneous outcomes rather than a single endpoint [6,11]. The pivotal hazard ratio for sGFAP relates to a combined sGFAP + sNfL exposure in a single cohort and is not, in itself, an sGFAP-specific estimate; the supporting sGFAP-only evidence derives from a small number of additional cohorts [31,32]. Several other domains also rest substantially on single cohorts (notably the digital step-count association), and the predictive validity of the digital SDMT for PIRA is unestablished [43,45,46]. Standardisation of MRI acquisition, serum assay platforms (including inter-centre Simoa calibration), and digital phenotyping algorithms remains incomplete [25,30]. Age confounds all four domains independently of MS [29]. Most contributing cohorts are predominantly White European; sGFAP and sNfL reference ranges may differ by ancestry, body mass index, and metabolic comorbidity, and existing thresholds cannot be assumed transferable to Latin American, Asian, or African populations without external validation [11,29]. Finally, and most importantly for the score itself, the MPS component selection is unvalidated, its equal-weighting assumption and integer ranges are placeholders that are mutually inconsistent as presented, and its risk tiers and action thresholds are illustrative; empirically derived weights from penalised survival models or federated networks should be the primary focus of prospective validation, and the score should not be used clinically until then [9,47].

## 6. Key Unanswered Questions in PIRA Biomarker Research

Several questions remain unresolved (Box 1) in PIRA biomarker research, including how to define sensitive composite endpoints, distinguish PIRA from relapse-associated worsening, and determine clinically meaningful thresholds for biomarkers such as sGFAP and PRLs. Future studies should validate multimodal biomarker scores across diverse populations and clarify the role of digital metrics, BTK inhibitor–related biomarker changes, and optimal modelling strategies for improving PIRA prediction and monitoring.

Box 1 **Key unanswered questions.**

**1. What is the optimal composite endpoint for PIRA in clinical trials?** EDSS-based CDW24 lacks sensitivity to early smouldering change. Should composite endpoints integrate retinal OCT, sGFAP, or digital ambulation metrics, and how should PIRA events be anchored to avoid conflation with RAW?

**2. Is the sGFAP–PIRA relationship causal or associative?** Elevated sGFAP is prospectively associated with PIRA, but its mechanistic role—reactive-astrogliosis driver versus downstream consequence of microglial activation—remains uncertain. Does suppressing sGFAP alter disability trajectories?

**3. What PRL count threshold should trigger closer review?** Studies use varying thresholds (≥1, ≥3, or ≥5 PRLs). Does a single PRL confer sufficient risk to alter management, or is a minimum burden required? Does rim activity matter beyond count, and how does lesion age affect interpretation?

**4. How should multimodal scores be validated across diverse populations?** Most longitudinal cohorts are predominantly White European. Age-normalised sGFAP and sNfL reference ranges may differ by ancestry, BMI, and comorbidity. Are existing thresholds transferable to Latin American, Asian, and African populations?

**5. Can digital health metrics complement clinic-based PIRA monitoring?** Passive accelerometry and digital SDMT may detect functional change, but regulators have not accepted continuous remote monitoring as a primary endpoint, and predictive validity for PIRA is unproven. What validation framework is needed?

**6. Does BTK inhibition normalise PIRA biomarkers?** Phase 3 trials of BTK inhibitors measure PIRA as a primary endpoint, but few pre-specify PRLs, sGFAP, or GCIPL as biomarker co-endpoints. Should platform trials incorporate multimodal PIRA biomarkers prospectively?

**7. What modelling architecture is optimal for the MPS?** Supervised survival models, federated neural networks, and foundation models offer different trade-offs between interpretability, cross-centre generalisability, and sample-size requirements. How should the score handle missing domain data?

## 7. Conclusions

PIRA and smouldering MS represent the central unmet therapeutic challenge in contemporary MS management. The contribution of this review is not new data but an explicit integration logic across four biomarker domains and a transparent, falsifiable roadmap for validating a composite, including competing-risks methodology, a biologically appropriate long-horizon endpoint, and external validation in under-represented populations. The proposed Multimodal PIRA Score is offered strictly as a hypothesis-generating scaffold—its component selection, weighting, and thresholds are provisional and, in places, deliberately left to be determined by data. Its near-term value, if validated, would lie in structuring prospective validation and in prognostic enrichment of trials targeting smouldering biology—not in guiding routine escalation of anti-inflammatory therapy. Realising this will require harmonised acquisition protocols, prospective multicentre cohorts with pre-specified relapse-independent endpoints, and rigorous model evaluation before any clinical adoption.

## Data Availability

No new data were created or analysed in this study. Data sharing is not applicable to this article.
